# Cardiac Tamponade During Congenital Diaphragmatic Hernia Repair in an Infant: A Case Report and Literature Review

**DOI:** 10.1002/ccr3.71463

**Published:** 2025-11-21

**Authors:** Pershia Davoodi Karsalari, Aylar Mohammadi, Noosha Samieefar, Gholamreza Ebrahimisaraj, Mehdi Sarafi, Nastaran Sadat Mahdavi, Parinaz Alizadeh

**Affiliations:** ^1^ Network of Interdiciplinarity in Neonates and Infants (NINI) Universal Scientific Education and Research Network (USERN) Tehran Iran; ^2^ Pediatric Urology and Regenerative Medicine Research Center Gene, Cell and Tissue Research Institute, Children's Medical Center, Tehran University of Medical Sciences Tehran Iran; ^3^ Pediatric Chronic Kidney Disease Research Center Gene, Cell & Tissue Research Institute, Children's Medical Center, Tehran University of Medical Sciences Tehran Iran; ^4^ Pediatric Surgery Research Center Research Institute for Children's Health, Shahid Beheshti University of Medical Sciences Tehran Iran; ^5^ Department of Anesthesiology, School of Medicine Mofid Children's Hospital, Shahid Beheshti University of Medical Sciences Tehran Iran; ^6^ Neonatal Health Research Center Research Institute for Children's Health, Shahid Beheshti University of Medical Sciences Tehran Iran

**Keywords:** congenital anomaly, congenital diaphragmatic hernia repair, intraoperative cardiac tamponade, neonatal cardiac arrest, neonatal intensive care, neonatal surgical complication, pericardiocentesis

## Abstract

Intraoperative cardiac tamponade is an exceptionally rare but life‐threatening complication during congenital diaphragmatic hernia repair in infants. In this case, pericardial effusion from inadvertent intravenous fluid extravasation led to cardiovascular collapse. Prompt recognition and pericardiocentesis were lifesaving, emphasizing vigilance when unexplained hemodynamic instability occurs during central or jugular venous access.

## Graphical abstract

Schematic depiction of congenital diaphragmatic hernia with abdominal organs displaced into the chest, accompanied by visual elements illustrating the key complications observed in this case: cardiac tamponade and pleural effusion. Created in BioRender. Davoodi Karsalari, P. (2025) https://BioRender.com/n8wws0e.

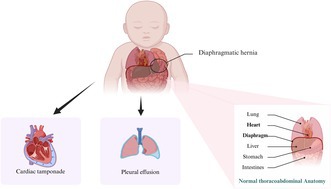

**Abbreviations**
CBC: complete blood count
CDH: congenital diaphragmatic hernia
CPR: cardiopulmonary resuscitation
CV line: central venous line
CVC: central venous catheterization
CXR: chest X‐ray
IUFD: intrauterine fetal demise
IUGR: intrauterine growth retardation
IV: intravenous
LTT: lymphocyte transformation test
mg/L: milligrams per liter
mL: milliliters
NICU: neonatal intensive care unit
PICC: peripherally inserted central catheter
SCID: severe combined immunodeficiency
TREC: T‐cell receptor excision circles

## 1 Introduction

Cardiac tamponade is a life‐threatening medical emergency characterized by the accumulation of pericardial fluid, blood, air, or pus in the pericardial space, leading to increased intrapericardial pressure, impaired cardiac filling, reduced cardiac output, and potentially fatal circulatory collapse [1]. It can result from various causes, including pericardial diseases, chest trauma, and iatrogenic complications such as surgical interventions and venous catheterization. Clinical manifestations typically include pulsus paradoxus, tachycardia, hypotension, dyspnea, and chest pain [2]. Pericardiocentesis remains the definitive treatment for relieving cardiac compression. Among the recognized causes of cardiac tamponade, central venous catheterization (CVC) is an important yet rare iatrogenic factor, particularly in neonates. Tamponade following CVC placement is often associated with catheter tip malposition or vascular perforation, and it occurs more frequently in cases of underlying anatomical abnormalities [3]. Notably, congenital diaphragmatic hernia (CDH), a rare developmental defect of the diaphragm, has also been linked to pericardial effusion in some cases [4]. Respectively, CDH leads to the herniation of abdominal organs into the thoracic cavity, resulting in pulmonary hypoplasia and pulmonary hypertension. It is classified based on its anatomical location, including posterolateral (Bochdalek), anterior (Morgagni), central, and rare mixed variants. Posterolateral CDH is the most common type, predominantly occurring on the left side [5]. In contrast, anterior CDH has been associated with significant pericardial effusion in infancy, though the exact mechanism remains unclear. Proposed explanations include lymphatic congestion due to thoracic duct compression, venous obstruction affecting the liver, or mechanical irritation from herniated tissues [4].

Here, we present the case of a neonate with a left‐sided posterolateral CDH who developed cardiac tamponade following surgical repair. The occurrence of tamponade in this setting is exceedingly rare, and while its exact cause remains uncertain, potential contributing factors include central venous catheterization and intraoperative hemodynamic shifts. This case underscores the need for vigilance in recognizing and managing such life‐threatening complications during CDH repair.

## 2 Case History/Examination

A male newborn was delivered via cesarean section at 38 weeks of gestation in a private hospital in November 2023. The mother was 40 years old (gravida 2, para 1) and delivered via repeat cesarean section. There were no problems during pregnancy and no family history. The birth weight was 3000 g and Apgar scores of 7 at 1 min and 9 at 5 min. During the initial examination in the delivery room, respiratory distress was seen and immediately endotracheal intubation was done. The baby was admitted to the neonatal intensive care unit (NICU). Intravenous (IV) cannula was secured, IV fluids in the form of crystalloids were started as maintenance.

## 3 Differential Diagnosis, Investigations and Treatment

**FIGURE 1:**
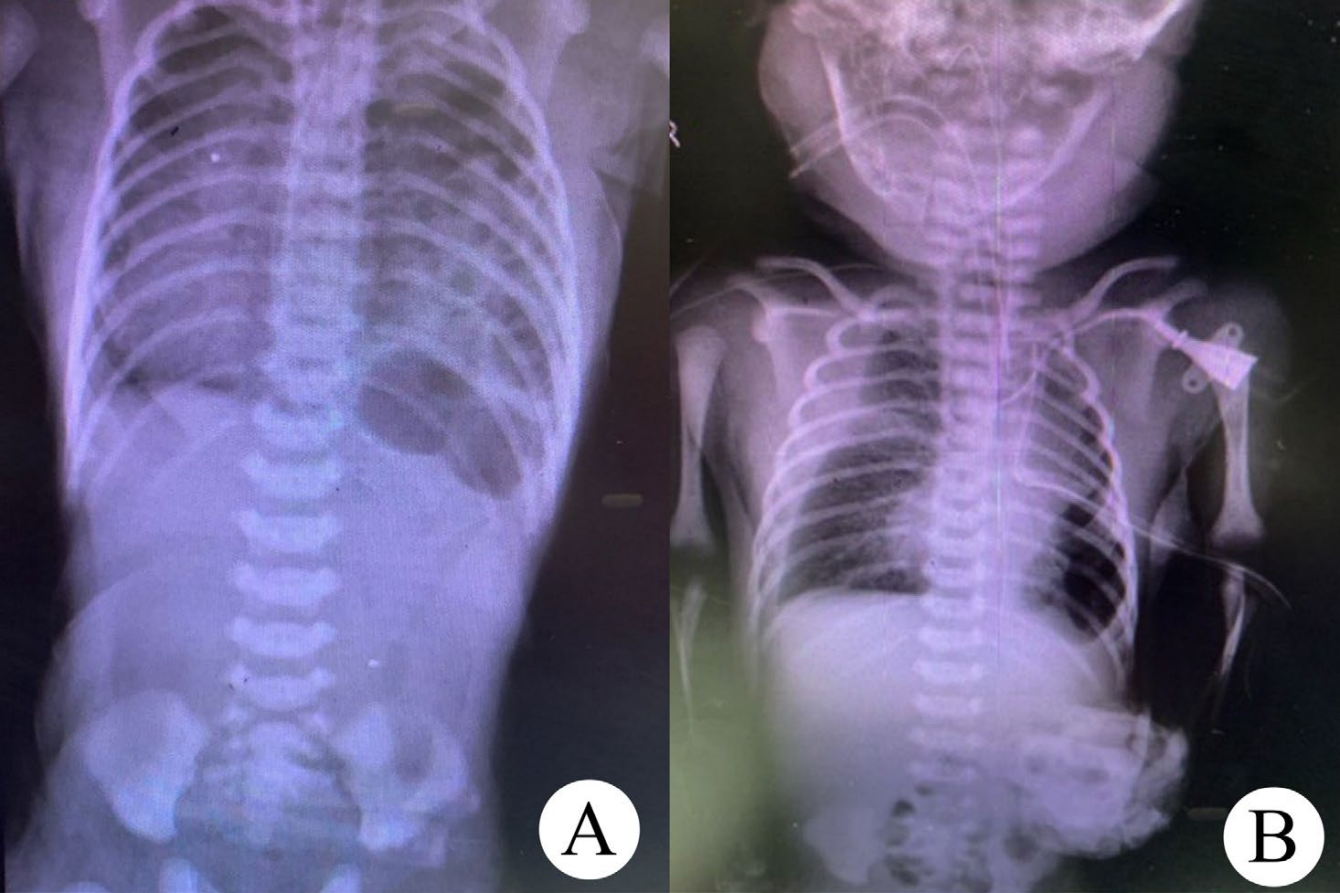
(A) Preoperative chest x‐ray (CXR) showing left‐sided congenital diaphragmatic hernia (CDH). (B) Postoperative CXR demonstrating right pleural effusion.

Although a chest x‐ray (CXR) performed on the first day suggested a left diaphragmatic hernia, the infant was kept stable and subsequently referred to our hospital on the third day of life. Upon admission, echocardiography revealed no pulmonary hypertension or cardiac anomaly, and the diagnosis of congenital diaphragmatic hernia (CDH) was confirmed (Figure 1). On the 4th day, surgery was performed to repair the hernia. A central venous line (CV line) was inserted into the left internal jugular vein, and a left chest tube was secured.

At the end of the surgery, the baby experienced sudden cardiac arrest. Cardiopulmonary resuscitation (CPR) was started. Due to abdominal distension and generalized cyanosis, abdominal compartment syndrome was initially suspected; the abdominal wall was opened, and the viscera were exteriorized and placed in a silo. However, the baby remained unresponsive to resuscitation despite repeated epinephrine doses and positive pressure ventilation.

Given the absence of cardiac sounds and persistent cyanosis, cardiac tamponade was suspected. Emergency pericardiocentesis was performed intraoperatively, yielding approximately 20 mL (mL) of clear fluid. Cardiac activity promptly resumed after the procedure, confirming tamponade. Biochemical analysis of the aspirated pericardial fluid revealed glucose levels identical to the 5% dextrose saline solution administered intraoperatively, **s**uggesting iatrogenic pericardial effusion due to intravenous fluid extravasation. No myocardial trauma was detected on postoperative echocardiography. No pericardial drain was left in place, and follow‐up echocardiography confirmed resolution of the effusion.

The baby was transferred to the NICU for close monitoring. Postoperative CXR showed right pleural effusion, and a right chest tube was inserted (Figure 1).

On the 6th day, the baby underwent closure of the abdominal wall and reduction of the viscera from the silo into the abdomen. Laboratory investigations during hospitalization showed normal complete blood counts (CBCs), while five sequential blood cultures obtained on subsequent days were positive for Gram‐negative bacteria (Acinetobacter, Serratia, Klebsiella, and Pseudomonas). Tracheal aspirate cultures were also twice positive for Gram‐negative organisms, and cerebrospinal fluid obtained by lumbar puncture was normal. The infectious disease team prescribed Amikacin and Meropenem treatment for 21 days. Immunology consultation was sought due to the recurrent sepsis and suspicion of severe combined immunodeficiency disease (SCID). The T‐cell receptor excision circles (TREC) test result was below the reference threshold, while CD flow cytometry was normal. A lymphocyte transformation test (LTT) was subsequently performed (Table 1). After 14 days of antibiotic treatment, blood cultures were negative. Several extubation attempts were unsuccessful, with final successful extubation achieved on Day 39.

**TABLE 1:** Lymphocyte transformation test (LTT) results.

Test	Patient's SI	Control's SI	Normal range
LTT‐PHA	7.8	6.3	≥ 3
LTT‐BCG	4.3	2.7	≥ 2.5

## 4 Conclusion and Results (Outcome and Follow‐Up)

After 55 days of hospitalization, the baby was discharged in good general condition. At follow‐up visits, the infant remained clinically stable, with normal echocardiography and no recurrence of pericardial effusion. Cardiac tamponade during CDH repair is an exceptionally rare but life‐threatening event, with very few cases reported in neonates. In this case, tamponade was confirmed intraoperatively and successfully treated with pericardiocentesis. Although the precise etiology remains uncertain, possible contributors include central or jugular venous line extravasation, surgical manipulation near the pericardium, and hemodynamic shifts during hernia reduction. This case underscores the importance of heightened vigilance for cardiac complications during CDH repair and demonstrates that prompt recognition and pericardial decompression are critical for survival.

## 5 Discussion

CDH remains a life‐threatening neonatal anomaly caused by defective diaphragm formation, leading to herniation of abdominal contents into the thoracic cavity [1]. While respiratory distress and pulmonary hypertension are well‐documented complications of CDH [6, 7], cardiac tamponade during or after CDH repair is exceedingly rare and scarcely reported.

In our patient, cardiac arrest occurred at the conclusion of surgical repair, and tamponade was promptly confirmed by pericardiocentesis, which drained approximately 20 mL of serum identical to the infused dextrose‐saline solution. This observation, combined with the absence of cardiac wall injury on postoperative echocardiography, strongly suggested iatrogenic infusion leakage into the pericardial space rather than a hemorrhagic or inflammatory effusion. The precise origin of this leakage remains uncertain, yet two mechanisms are likely: inadvertent perforation of the cardiac or pericardial wall by a jugular venous or central line, or extravasation through microtears in the vessel wall induced by pressure or movement during thoracic manipulation [3, 8, 9]. A similar mechanism was described by Butt et al., who reported cardiac tamponade secondary to pericardial infusion from a CVC in an adult patient. Although their case involved an older individual, it underscores the universal risk of fluid extravasation when the catheter tip lies close to or within the cardiac silhouette [3]. In neonates, the risk is even greater due to the thin atrial wall and small pericardial volume, meaning even a few milliliters of infused fluid can produce critical tamponade physiology within seconds [2].

A recent report described a 10‐day‐old preterm neonate who developed cardiac tamponade secondary to a peripherally inserted central catheter (PICC). The infant suddenly presented with hypotonia, apnea, hypoxia, hypotension, and bradycardia. Echocardiography revealed severe pericardial effusion, and urgent pericardiocentesis along with catheter removal resulted in rapid stabilization. This case mirrors the mechanism observed in our patient, emphasizing that even correctly positioned neonatal central lines or PICCs can cause life‐threatening pericardial effusions, highlighting the need for vigilant monitoring and preparedness for immediate intervention [10].

Physiologically, the hemodynamic shifts inherent to CDH repair may also contribute. Rapid decompression of the thoracic cavity alters venous return and myocardial filling pressures, while surgical manipulation near the pericardium can increase capillary permeability or cause microscopic tears [11]. These factors may act synergistically with a misplaced or traumatized venous line to precipitate pericardial fluid accumulation. Thus, both surgical and iatrogenic factors must be considered [3].

During the intraoperative arrest, abdominal compartment syndrome was initially suspected due to generalized cyanosis and distension, prompting decompression and silo placement. However, despite these interventions and repeated epinephrine administration during CPR, there was no return of cardiac activity until pericardiocentesis was performed. The immediate restoration **of** cardiac rhythm after pericardial drainage confirmed tamponade as the primary cause of arrest.

The acute management of cardiac tamponade in neonates hinges on rapid recognition and decompression. In emergent intraoperative settings where imaging is unavailable, diagnosis relies on clinical judgment. Sudden cardiovascular collapse unresponsive to standard resuscitative measures should prompt urgent pericardial aspiration [10]. In this case, the swift decision to perform pericardiocentesis proved life‐saving. Postoperative recovery was uneventful, and no recurrent effusion was observed on follow‐up echocardiography.

In summary, this case highlights a rare but catastrophic event: cardiac tamponade due to the infusion of intravenous serum into the pericardial space during CDH repair. Even in the absence of direct cardiac injury, such complications may arise from subtle catheter malposition or vessel wall microperforation. Surgeons and anesthesiologists should maintain a high index of suspicion when unexplained hemodynamic collapse occurs, and immediate pericardiocentesis remains the critical step in acute management. Vigilant line placement, frequent monitoring, and readiness for emergency decompression are essential safeguards during neonatal CDH surgery.

A review of the literature revealed multiple reports of CDH in older children and adults associated with cardiac complications (Table 2).

**TABLE 2:** Summary of case reports on congenital diaphragmatic hernia (CDH) accompanied by cardiac manifestations.

Author/year	Country	Age/sex	Clinical manifestations	Radiological investigations	Lab results	Operative findings	Follow‐up
Shin et al. (2020) [12]	South Korea	13 years old, male	Experiencing abdominal discomfort in the upper left quadrant and episodes of nausea and vomiting within the last 3 days before hospitalization	The first chest x‐ray revealed a hemithorax obscured by opacity, accompanied by a contralateral mediastinal shift, resembling pleural effusion. Following the initial diagnosis of pleural effusion, a chest tube thoracotomy was conducted, resulting in the drainage of substances resembling food. Chest CT scan detected CDH and iatrogenic gastric perforation caused by chest tube insertion.	White blood cell—11,990/mL, hemoglobin— 17.3 g/dL, high‐sensitivity C‐reactive Protein—11.03 mg/L platelet—450,000/mL	The procedure revealed a substantial hernial gap on the left posterolateral section of the diaphragm, measuring 10 by 5 cm, allowing various organs to displace into the thoracic cavity.	Improved
Zarkesh et al. (2022) [10]	Iran	10‐day‐old, male, preterm	Sudden hypotonia, apnea, hypoxia, hypotension, bradycardia on day 10 post‐PICC	Chest X‐ray: PICC in appropriate position; echocardiography: severe pericardial effusion with tamponade		Pericardiocentesis performed; PICC removed	Dramatic improvement, stabilized vital signs, resolved tamponade
Salmanian et al. (2014) [13]	USA	37 years old pregnant with a female fetus	At 31 weeks of gestation, the patient's fetus displayed a sizable CDH predominantly on the right side, leading to a considerable portion of the fetal liver protruding into the chest cavity. This condition resulted in significant ascites, elevated intrathoracic fluid pressure, mediastinal deviation, hydrops fetalis, and physiological changes reminiscent of tamponade, all of which compromised cardiac function.	During routine first trimester ultrasound screening at 12 weeks of gestation, suspicions arose regarding a cystic adenomatoid mass in the right fetal lung. A subsequent ultrasound scan at 15 weeks confirmed the presence of a CDH on the right side. At 27 weeks, fetal MRI revealed a sizable right‐sided diaphragmatic hernia, encapsulating a significant portion of the right hepatic lobe, a small amount of small bowel, and fluid. The right lung was compressed upwards and towards the middle, while the left diaphragm remained intact. Although the mediastinum and heart shifted to the left, no signs of pleural effusion or hydrops were observed. By 31 weeks, a follow‐up ultrasound showed worsening ascites, alongside a small pericardial effusion, skin edema, and severe polyhydramnios. Additionally, the fetal echocardiography revealed bilateral diastolic collapse of the atrial roof attributed to compression from fluid masses.		Initially, a peritoneal shunt was attempted. Following the placement of the peritoneal amniotic shunt, it was determined that the liver had effectively obstructed the diaphragmatic hernia and impeded the intrathoracic fluid drainage. Subsequently, a second shunt was inserted into the fetal chest fluid, leading to immediate and subsequent drainage of the intrathoracic fluid, which elevated pressure and resulted in an immediate improvement in cardiac function	Improved
Morgan et al. (2020) [14]	USA	63 years old, male	The patient experienced postprandial abdominal pain, inability to pass gas, and repeated vomiting	The first abdominal and pelvic CT scan with contrast conducted at another medical facility indicated a singular expansion of the stomach. Following a fall, a subsequent CT scan was performed, uncovering bowel loops inside the pericardial sac, pericardial effusion, and herniated bowel pneumatosis. During the fall, the patient experienced an SVT episode, leading to resuscitation, intubation, and subsequent ordering of a CT scan and echocardiogram. The later echocardiogram indicated the presence of fluid accumulation and signs of cardiac tamponade.	Leukocyte = 14 (indicating leukocytosis)	The diaphragmatic hernia was observed and repaired.	Improved
Matsushita et al. (2007) [15]	Australia	57 years old, male	The patient had been experiencing worsening chest pain and shortness of breath over several years. He did not report any abdominal symptoms, but his superficial veins in the upper limbs were significantly enlarged.	The chest CT scan showed herniation of abdominal contents into the anterior mediastinum through a left‐sided diaphragm defect, compressing the right ventricle. Additionally, a large sliding hiatal hernia with abdominal contents in the posterior mediastinum was observed on CT.		The anterior mediastinum was entered, uncovering a substantial hernia sac emerging from the left foramen of Morgagni. Extending up to the sternal notch, the sac contained a majority of the transverse colon, small intestine, and a significant amount of omentum.	Improved
Manson et al. (2017) [16]	UK	30 years old, female	The patient primarily reported increasing pain in the upper abdomen, with their medical background limited to an episode of dyspepsia 5 years earlier.	CT scan revealed an isolated herniation of the stomach into the chest cavity. Esophagogastroduodenoscopy showed slight gastric mucosal bleeding but no signs of ischemia. An immediate thoracic CT scan confirmed a Bockdalek hernia through the left hemi diaphragm's posterolateral aspect, with both stomach and spleen experiencing strangulation.	Oxygen desaturation, Significant respiratory acidosis	The results of the subsequent laparotomy revealed gastric volvulus leading to ischemia in both the stomach and spleen. Adhesions around these organs suggested chronic herniation.	Improved
Pribadi et al. (2015) [17]	Indonesia	34–35 weeks		The ultrasound revealed dextrocardia along with other cardiac abnormalities. The diagnosis was Bockdalek CDH, with the heart shifted to the right and mild hypoplasia of both ventricles		The diagnosis was confirmed as Bockdalek CDH.	Improved
			Intrauterine growth Retardation (IUGR)	The ultrasound revealed significant anterior positioning of the heart, along with a fluid‐filled mass pressing anteriorly from the posterior side of the heart. It also showed hypoplasia of the right ventricle with tricuspid valve stenosis, dilation of the left ventricle, and abnormalities related to mitral valve regurgitation. Additionally, polyhydramnios was noted. The diagnosis was Bockdalek CDH.			Intrauterine fetal demise (IUFD)
				The ultrasound revealed the heart shifted towards the left, with a cardiothoracic ratio below normal. A cystic mass was observed in the anterior thoracic cavity, specifically in the left parasternal area. The diagnosis was Morgagni CDH.		The diagnosis was confirmed as Morgagni CDH.	Survived

## Author Contributions

**Pershia Davoodi Karsalari:** conceptualization, data curation, visualization, writing – original draft, writing – review and editing. **Aylar Mohammadi:** writing – original draft. **Noosha Samieefar:** writing – review and editing. **Gholamreza Ebrahimisaraj:** methodology. **Mehdi Sarafi:** methodology. **Nastaran Sadat Mahdavi:** methodology. **Parinaz Alizadeh:** supervision, writing – review and editing.

## Disclosure

Patient perspective: The family was relieved once the accurate diagnosis and effective treatment were initiated, and they appreciated the dedication of the multidisciplinary care team in finally resolving the persistent symptoms.

## Ethics Statement

The authors have nothing to report.

## Consent

Written informed consent was obtained from the patient's guardians for the publication of this case.

## Conflicts of Interest

The authors declare no conflicts of interest.

## Data Availability

The authors have nothing to report.
